# The Mortality of Periviable and Extremely Premature Infants and Their Impact on the Overall Neonatal Mortality Rate

**DOI:** 10.1038/s41598-020-59566-3

**Published:** 2020-02-12

**Authors:** Horacio S. Falciglia, Ronald C. Merkel, Vickie Glover, Kimberly A. Hasselfeld, W. Kim Brady

**Affiliations:** 1 Perinatal Institute, Section of Neonatology, Perinatal & Pulmonary Biology, Cincinnati Children’s Hospital Medical Center, Cincinnati, Ohio USA; 2 Department of Obstetrics and Gynecology, Good Samaritan Hospital, Cincinnati, Ohio USA; 3 TriHealth Hatton Research Institute, Good Samaritan Hospital, Cincinnati, Ohio USA; 4 Department of Orthopaedic Surgery, University of Cincinnati College of Medicine, Cincinnati, Ohio USA

**Keywords:** Physiology, Neonatology

## Abstract

To investigate mortality in periviable neonates ≤23 weeks gestational age and calculate its impact on overall neonatal mortality rate over a 12-year period (1998–2009). Verify if periviable mortality decreased in the period (2010–2015). Retrospective review. Neonatal mortality rate per 1000 live births was 11.4. Three hundred forty-nine live birth infants weighed ≤500 g and 336 died. Their proportion to the total neonatal mortality rate was 48.6%; out of 298 periviables 146 (43%) were ≤20 weeks gestational age. In 269 (80%) we could not determine the cause of death. Two hundred ninety-seven neonates (88.3%) died in the delivery room. Sixteen (5%) had an autopsy. Neonatal mortality rate from periviability was 96.2% and constituted half of the overall rate in the period (1998–2009). There was not significant reduction of periviable mortality between 2010 and 2015. Current live birth definition and a reporting system that considers a 100 g periviable live birth infant as a neonatal death has placed Ohio and the United States at a significant disadvantage compared to other countries using different reporting systems.

## Introduction

Cincinnati, Ohio has high neonatal and infant mortality rates. In Hamilton County, which includes Cincinnati, the infant mortality rate is among the worst 10% in the United States of America (USA)^1^. The three leading causes of infant deaths in Hamilton County, Ohio were preterm births, birth defects and sleep-related deaths. About half of all local babies born before the end of the second trimester do not survive. This extreme prematurity is the leading cause of infant death in Hamilton County^1^.

Gestational age (GA) and weight are the most important factors in predicting survival rates of premature infants, which have improved due to advances in both prenatal and postnatal care. However, in spite of extraordinary medical and economic efforts, survival remains dismal^2^.

A periviable birth according to the Society for Maternal-Fetal Medicine, the Eunice Kennedy Shriver National Institute of Child Health and Human Development, the Section on Perinatal Pediatrics of the American Academy of Pediatrics, and the American College of Obstetrician and Gynecologists is defined as a delivery that occurs from 20 0/7 weeks to 25 6/7 weeks of gestation^3^.

In the USA all deliveries (excluding termination of pregnancy) are registered with either a Certificate of Live Birth or a Certificate of Fetal Death^4,5^. There is a lack of uniformity and standardization on the issues of stillbirth and live births (LBs). For instance, different definitions of stillbirth are used in different states and different countries. In the USA both the Centers for Disease Control and Prevention and the American College of Obstetricians and Gynecologists endorse a definition of stillbirth that uses a GA of 20 weeks and weight ≥350 grams (g), while the World Health Organization (WHO) uses reporting criteria of ≥28 weeks GA and a birth weight of 1000 g. The same lack of standardization occurs with LBs. In the state of Ohio, LBs include any infant who has taken a breath or showed any other evidence of life, such as beating of the heart, pulsation of the umbilical cord, or definite movement of voluntary muscles, regardless of whether the umbilical cord has been cut or the placenta is attached (H.B Number 790, Section 3705.01 (A))^5^. The Ohio and USA criteria disregard GA, weight or how long the infant lived. In some countries of the European Union and Scandinavia, any product of conception delivered prior to 23 weeks GA is not considered a LB, and as such is not counted in survival statistics^6^.

The purpose of this project was to determine the impact of the periviable mortality on the total neonatal mortality rate (NMR) at Good Samaritan Hospital (GSH) Cincinnati, Ohio from 1998–2009. With advances in prenatal and pediatric care, there appears to be some indication that “a plateau in survival may have been reached”^7^, and that the extremely preterm neonate at the limits of viability and periviable neonates constitute the majority of the current NMR. Therefore our aims in this study were:

1. To determine the overall annual NMR and autopsy rate at GSH from 1998–2009;
2. To develop an annual adjusted NMR that excludes neonates born ≤23 0/7 weeks GA and weighing ≤500 g at birth;
3. To calculate the proportion of neonatal deaths from periviable neonates in the period 1998–2009 and to verify if periviable mortality decreased in the period 2010–2015;
4. And to determine the cause of death in periviable neonates ≤23/07 weeks and ≤500 g in weight.

Despite information available at Mortality Committee reviews, including maternal, family, obstetrical history, placental pathology and autopsy results, we hypothesized that the periviable infants would contribute to half of the NMR and that the cause of death would be unknown in 90% of the cases. We believed that a better understanding of GSH’s neonatal and infant mortality rate would enable physicians to better counsel families of the very preterm neonate and provide understanding of what factors contribute to the mortality rate of the most at-risk infants.

## Subjects and Methods

The Institutional Review Board at the GSH in Cincinnati, Ohio waived the need for written informed consent and approved this study. GSH is an urban general hospital with a large maternity service and a Level 3 Neonatal Intensive Care Unit (NICU). Additionally, it is serviced by Cincinnati Children’s Hospital Medical Center (CCHMC) staff. During the 1998–2009 and 2010–2015 periods, GSH had the largest number of deliveries in Ohio at 6500–7000 births annually. Cincinnati GSH has been the number one ranking hospital in Ohio for delivering infants^8^, for several years in a row. Data in this study was obtained from a retrospective review of the minutes from the GSH Neonatal Mortality and Morbidity Committee to identify the cause of death as determined by the following: (a) presumed clinical cause of death by the primary care neonatologist and/or the Committee members; (b) autopsy and placenta examination when available. Data was also collected from (c) maternal, neonatal, and delivery room resuscitation records; (d) the Vermont Oxford Network database for GSH patients; and (e) data obtained from the CCHMC Perinatal Outreach Project and Cradle Cincinnati. Cradle Cincinnati is a collaborative between civic leaders, health departments, maternity hospitals and other institutions whose mission is to reduce infant mortality^1^.

There was no patient contact as this was a retrospective record review. The study presented minimal risk to patients and it was limited to the potential for accidental release of protected health information. While patient identifiers, including names and medical record numbers, were accessed and recorded, they were used solely to facilitate review of records from different data sources and to allow auditing to ensure accurate and complete records. Data entry fields were coded to prevent accidental download of the information. Further, patients were assigned a unique identifier by the REDCap system to prevent the re-identification of patient records^9^. Upon completion of data collection, identifiers were removed and the key was held within the Perinatal Research Center. The database had two levels of security: system level access was controlled by password, and database access was controlled by invitation.

Definitions: Chorioamnionitis with or without preterm Premature rupture of Membranes (PPROM) was defined as maternal fever without a source, fetal tachycardia and a maternal white blood count ≥15,000/mm and maternal uterine tenderness. Multiple gestation: two or more fetuses were documented at any time during pregnancy which resulted in the birth of the infant. Incompetent cervix included the inability of the cervix to retain a pregnancy in the second trimester, in absence of uterine contractions; presenting with painless cervical dilatation, PPROM, with subsequent rapid delivery of often a periviable neonate. Placenta abnormalities included: velamentous insertion of umbilical cord, circumvallate placenta, hydropic and molar placenta. Substance abuse included cocaine, heroin and amphetamines. Respiratory Distress Syndrome (RDS) was defined as a PaO_2_ < 50 mm Hg in room air, supplemental oxygen to maintain PaO_2_ > 50 mmHg and a chest-x-ray consistent with RDS, reticulogranular appearance of the lung and air bronchograms. Bacterial sepsis: bacterial pathogen was recovered from a blood and/or cerebrospinal culture. Lethal anomalies were considered lethal or life threatening if the anomaly was either (1) the primary cause of death or (2) treated prior to discharge with specific surgical or medical therapy to correct a major anatomic anomaly of the Central nervous system, heart, gastrointestinal, genitourinary and pulmonary systems. Intraventricular Hemorrhage (IVH): grade 3: intraventricular blood, ventricular dilation; grade 4 intraparenchymal hemorrhage diagnosed by ultrasound, Cranial Tomography, or Magnetic Resonance Image (MRI).

One of the authors (WKB) reviewed obstetrical medical records and determined the best estimate of GA for neonates ≤500 g using the following method: (1) last menstrual period (LMP) alone; (2) 1^st^ trimester ultrasound (US) alone; (3) 2^nd^ trimester US alone; (4) 3rd trimester US alone; (5) 1st trimester US consistent with LMP; (6) 2nd trimester US consistent with LMP; (7) 3^rd^ trimester US consistent with LMP; and or (8) neonatal exam by neonatologist using the New Ballard score^10^, expanded to include extremely premature infants for 20–23 0/7 weeks gestation.

NMR was defined as the number of deaths during the first 28 completed days of life/live births per 1000.

The definition of periviability at the time we wrote the protocol for this research project (4/10/2011) was any LB up to ≤23 weeks 0/7 (23 completed weeks gestation), reported to the state of Ohio as a neonatal death or as a survivor.

## Results

Among 73,547 total LBs there were 699 neonatal deaths and the autopsy rate was 13.3%. The average unadjusted NMR per 1,000 LBs was 11.4. The average adjusted NMR was 4.9, (adjusted for lethal anomalies and weight ≤500 g). Of 349 LBs weighing ≤500 g, the mortality was 96.3% (n = 336). The proportion of neonatal deaths ≤500 g to total neonatal mortality was 48.1% (336/699).

Gestational age as described in the methods was 15–17 weeks in 37 neonates, 18–20 weeks in 109, and 21–23 0/7 weeks in 152; in addition we reported to the State of Ohio 38 neonates 24–25 0/7 weeks GA with weight ≤500 g. The weight and number of infants are described in Table 1.

**Table 1:** Weight in grams in dead periviable neonates ≤500 g.

Grams	n (%)
30–100	11 (3%)
101–200	42 (13%)
201–300	63 (19%)
301–400	98 (29%)
401–500	122 (36%)
Total	336 (100%)

Among the neonatal deaths ≤500 g, 257 mothers (77%) received prenatal care, 47 (14%) did not receive prenatal care, and for two mothers it was not documented. Two-hundred eighty-eight mothers (86%) had a vaginal delivery while 46 (14%) were delivered by Cesarean section. In two cases delivery mode was not documented. Among all maternal complications described in Table 2, chorioamnionitis in 217 (65%) mothers, significantly contributed to periviability (Chi-square p < 0.05,Yates correction 56.006).

**Table 2:** Maternal complications associated with periviability (n = 336).

Diagnosis	n (%)
Chorioamnionitis*	217 (65)
Multiple Gestation	130 (39)
Incompetent Cervix	73 (22)
Abruptio Placentae	62 (18)
Substance Abuse	30 (9)
Preeclampsia or HELLP	27 (8)
Diabetes	14 (4)
Placental Abnormalities	19 (6)
Others: includes *in vitro* fertilization, protein S deficiency, twin-twin transfusion syndrome, sexually transmitted disease, isoimmunization, sickle cell disease, lupus erythematous.	59 (18)

*We used the Chi-Square statistic to examine the proportion of neonates with chorioamnionitis versus those without. The Chi-Square statistic with Yates correction was 56.006. The p-value was significant at p < 0.05.

There were substantially more males (59%) in the cohort population of dead neonates, but male mortality was not different than in females (Chi-square p value 0.69); racial profile among dead neonates, 73% Caucasians, reflected our predominant white inborn population at GSH of 75%. On the contrary in the rest of the city of Cincinnati, African Americans had a NMR of 10.54 per 1000 LBs, 2 and1/2 times higher than the 4.06 per 1000 LBs in white infants during the study period^11^.

Causes of death in the periviable neonates are listed in Table 3. Mortality Committee examined the proportion of 87 (20%) neonates with a known cause of death versus 269 (80%) of neonates where the diagnosis was uncertain beyond periviability and it was significant that no cause of death could be determined in the majority of the neonates (Chi-square with Yates correction was 184.0506, p < 0.05)

**Table 3:** Cause of death in periviable neonates ≤500 g.

Diagnosis	n (%)
Respiratory Distress Syndrome	29 (9%)
Bacterial Sepsis	25 (7%)
Lethal Anomalies	17 (5%)
Intraventricular hemorrhage, grade 3 or 4	7 (2%)
Pulmonary hemorrhage	5 (2%)
Pneumonia	2 (1%)
Necrotizing enterocolitis	2 (1%)
Uncertain diagnosis beyond periviability*	269 (80%)

*We used the Chi-Square statistic to examine the proportion of neonates with a diagnosis for their death versus those where the diagnosis was uncertain beyond periviability. The Chi-Square statistic with Yates correction was 184.0506. The p-value was significant at p < 0.05.

**Figure 1:**
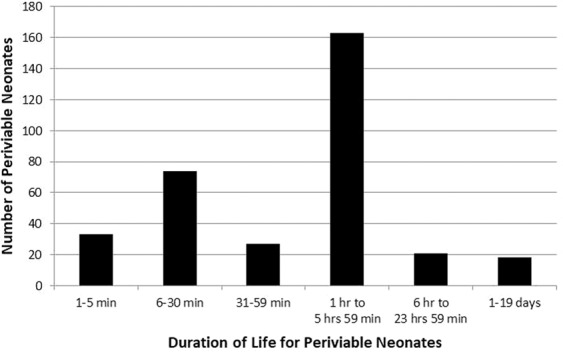
Duration of life for periviable neonates in the study.

Among dead premature neonates ≤500 g (n = 336), 297 (89%) died in the delivery room within 6 hours after delivery (Fig. 1). According to family wishes and or medical judgment, out of 349 LBs only 38 neonates (11%) received postnatal resuscitation and then they were transferred to the Level 3 NICU where 25 died and 13 survived. Among the dead neonates ≤500 g, in 291 (87%) autopsy was not requested and/or not discussed with the family. In 45 neonates (13%) autopsy was requested and in only 5% (16 of 336 deaths) the parents authorized an autopsy and it was performed. Family refused the autopsy in 29 infants. The average NMR < 500 g in the period 2010–2015 was 94,3% and not significantly different (P value = NS) to the period 1998–2009. In 2010 was = 95,8; 2011 = 93,3; 2012 = 95,2; 2013 = 91,8; 2014 = 93,8 and 2015 = 95,6.

## Discussion

The results of our study reflect the lack of a clear definition of periviability in our hospital during the study period 1998–2009. Following state of Ohio maternity regulations, we had to report 146 LB’s neonates of a gestation ≤20 weeks who were classified as LB’s and died within six hours of birth. In addition, our study reflects the general lack of clear guidelines regarding obstetric interventions for threatened and imminent periviable births during the study period. Other local hospitals in the Cincinnati area suffered similar problems. In our study there were no clear guidelines on neonatal assessment for resuscitation. Resuscitation of the infant was decided on a case-by-case basis, sometimes by the physicians caring for the infant, sometimes following parents request, other times by both, doctors and parents. This lack of consensus is also seen at a national level. A national survey of obstetricians’ attitudes toward periviable intervention^12^ demonstrated that there are personal and institutional factors that may influence obstetrical counseling and decision-making. Respondents reported institutional cutoffs of 23 weeks for resuscitation (34%) and 24 weeks for Cesarean sections (35%). At 23 weeks, two thirds ordered steroids, 43% recommended Cesarean section and 23% offered induction^12^. This lack of agreement is confirmed by a recent report^13^ on practices and education surrounding anticipated periviable deliveries. Neonatal-Perinatal Medicine and Maternal-Fetal Medicine fellowship programs reported different, often asynchronous, practices and fellow education regarding antenatal counseling and resuscitation at periviability.

Our data also needs to be interpreted within the frame of USA data. The USA lags on survival and during the time our study was conducted the USA ranked near the bottom among modern nations, better only than Latvia^14^. Part of the difference lies in the reporting system of what constitutes a LB and what is a reportable neonatal death. More recently in 2013, a study by Lorenz, *et al*.^15^ showed the USA infant mortality rate was reported the highest among developed countries, ranking 29^th^, higher only than Mexico and Turkey.

Knowledge of survival rates aids family counseling and guides treatment decisions. Infants born too early face lifelong challenges however, the first and greatest challenge is survival. Extremely low birth weight infants are often at the very limits of viability and despite advanced levels of care provided cannot survive. Kamath, *et al*. found with advances in maternity and neonatal care, neonatal survival for Colorado infants with a birth weight of 750–1500 g has shown significant improvement for the years 1997–2003 when compared to the 1991–1996. However, for infants with a birth weight below 750 g, survival rates have not increased^16^. Similarly, Fanaroff, *et al*. found no improvement in survival rates without long-term morbidity from 1995–2002 despite a push toward regionalization and a preponderance of evidence that regionalization of high risk births at a Level III care facility provides the greatest chance of survival^17,18^. GSH is a level III Regional Perinatal Center.

Neonates with birth weights <750 g have high rates of RDS, chronic lung disease, bacterial sepsis, NEC and IVH^19^. Interventions to improve survival include antenatal steroids and antibiotics, Cesarean section, and respiratory treatments including surfactant therapy and mechanical ventilation^18,20–22^. Each additional week of gestation provides a greater chance of survival, increasing from 2–6% at 22 weeks of gestation to 49–72% at 25 weeks^20,21^. Long term outcomes for these babies remain poor and studies suggest aggressive efforts may prolong life for only a few months and result in children with serious morbidities^23–25^.

In the majority of the neonatal deaths in our study, 257 mothers (77%) received prenatal care of two or more visits, so we don’t consider prenatal care a factor in our high NMR.

Two hundred ninety-seven (89%) died in the delivery room within six hours of being born. NMR ≤ 500 g represented 48.1% of the overall NMR during 1998–2009 and it confirmed our main hypotheses. RDS, sepsis and lethal anomalies were the leading known causes of death in this study, however, they represented only 20% of the neonates; in the majority of the dead neonates (80%) we could not determine a cause of death primarily due to the lack of an autopsy.

It is generally accepted that autopsy results in enhanced knowledge of the cause of death. The low autopsy rate in our institution is not unique and it has been confirmed by the Canadian investigators in Auger, *et al*.^26^. In their study, autopsy rates declined by 29% for stillbirths and 36% for infant deaths during the period 1981–2015 in Quebec, Canada. They concluded that greater use of the autopsy has a potential to minimize the number of stillbirths with an undetermined cause of death, and may be helpful for prevention. A major setback to the rate of autopsy was dealt in 1970 when the Joint Commission on Accreditation of Hospitals and Accreditation Council for Graduate Medical Education eliminated autopsies as a requirement for teaching hospitals. Another important cause of reduction in autopsies at our institution could be the reluctance of our medical staff to recommend it, due to medical legal concerns. Sometimes the autopsy uncovers missed clinical diagnoses and often there is also reluctance by the families due to their misconceptions about the autopsy. They may hold the belief that the cause of death is known from previous diagnostic testing and recognition that an autopsy cannot save the child^27–30^. Also, in many cases, obstetricians and pediatricians may be ignorant of the existence of a partial autopsy, which could clarify the cause of death. The author (HSF) has unpublished cases of congenital pneumonia in patients with history of chorioanmionitis where the diagnosis was made with a simple lung tissue culture. Some researchers have found that parents are more likely to consent to a less invasive method than the full autopsy^31,32^.

The need for an autopsy is also supported by Barton and Hodgman’s study^33^. A total of 111 infants between 300–1000 g at birth who died were autopsied and included placental examinations and autopsy cultures. Infection was the most common cause of death in over 50% of the deaths. Infection, as congenital pneumonia was significantly underdiagnosed clinically, with most of these deaths attributed to immaturity or RDS^33^. It is possible that in our study, where there was a high rate (65%) of chorioamnionitis, the performance of an autopsy could have uncovered as well, unrecognized congenital pneumonia as a cause of death.

Other factors that may have played a role against ordering the autopsy in our patients included the cost of the autopsy. The non-billable cost of $1,800–$3,000 is not covered by insurance.

Autopsy has been able to determine rare genetic diseases leading to perinatal deaths including chromosomal anomalies (i.e. Trisomy 13, Pallister Killian Syndrome tetrasomy 12 p), deletion 22q11 syndrome, and inborn errors of metabolism^32^; autopsy also discover cardiac arrhythmias as a cause of death^33^. For the infants who die without a diagnosis, there are advances in genomic technology such as the “molecular autopsy” that in the future can be incorporated^34^.

Regarding the poor NMR in the USA, a report from Mikkel Oestergaard and the WHO^35^ is being cited as an indictment of the USA Health care system with the USA ranking 41^st^ in NMR; the authors quote ‘however infant and NMR are complex multifactorial end-points that oversimplify heterogeneous inputs, many of which have no relation to health care at all”. Underreporting and unreliability of NMR data from other countries undermine any comparisons with the United States. Also gross differences in the definition of LB invalidate comparisons of early NMR. The USA, including Ohio, strictly adheres to the WHO definition of a LB which we previously described, however this definition is irrespective of the duration of pregnancy and weight. On the contrary some European Union countries use 23 or 24 weeks as a cut off point for recording demise as a neonatal death. Other countries including 27 highly developed nations in Western Europe^6^ show significant differences in reporting LBs. Although a majority of countries, 20 out of 27 WHO participating countries make use of the WHO signs of life criteria required for defining a LB, some of them like Poland,ex-Czechoslovakia and former USSR still impose additional viability criteria such as a minimum life duration after birth, required for official registration as a LB. Also a minimum birth weight of 500 grs or greater has been additional requirements for a LB registration in the Netherland, France and Romania; all of these requirements are leading to under-registration of LBs in Europe and in part explains the disparity with the USA.

In summary, the NMR at GSH in Cincinnati, Ohio was deceptively elevated because of a local and national reporting system^5^ that considered during the study period a 20 weeks GA 100 g periviable birth, a LB and it was counted as a neonatal death. One hundred forty-six infant deaths comprised 43% of the total deaths and they were ≤ 20 weeks GA. In over 80% of the neonatal deaths there was not a recognizable cause of death and this percentage was a very close rate to our hypotheses of 90%; also the findings of this study confirms our main hypotheses that periviability contributes to half of the NMR. The low rate of the periviables’ autopsies in our hospital also contributed to these findings. The lack of standardization in definitions of LBs at the national and international level is also a major reason for the poor USA ranking performance in NMR. In the future, the USA and other countries need to have a uniform, standard and consistent definition of LBs if we want to be able to compare “apples to apples” and make rankings useful tools among countries to reduce neonatal mortality. In our opinion, the time for standard definition and improved reporting has arrived. At the time of this writing (5/11/2017) Cradle Cincinnati announced that Hamilton County Cincinnati infant mortality is improving faster than the state of Ohio and the nation yet remains very high”^11^. From 2012–2016 Hamilton County had a historically low infant mortality rate with 8.9 deaths for every 1000 LBs. This 20% drop in mortality was twice the rate of improvement at the national level and the fastest in the state of Ohio. However, our rate of neonatal deaths remains much higher than the national average of 5.8 deaths per 1000 LBs. The recent review of our periviable NMR ≤ 500 grs from 2010 through 2015 has been 94.3%, very similar to the 96.2% of the 1998–2009 period^1,11^. These data support the findings of this study and our message to increase the rate of the autopsies.

## Data Availability

Data is available from author and Principal Investigator HSF.
